# Hospital Needs and Hospital Appeals

**Published:** 1923-12

**Authors:** 


### HOSPITAL NEEDS AND HOSPITAL APPEALS.

### HOSPITALS WITH 150 BEDS AND UPWARDS.

**BROMPTON HOSPITAL FOR CONSUMPTION AND DISEASES OF THE CHEST.**—With its sanatoria and convalescent home at Chobham Ridges, this hospital has nearly 500 beds, all of which are constantly occupied. During 1922, 2,943 in-patients and 35,688 out-patients received treatment at this institution. The annual expenditure is over £75,000, of which only £5,000 is assured. Donors of £52 10s. and annual subscribers of £5 5s. become Governors, and may recommend one in-patient and eight out-patients every year. The Secretary, Mr. Frederick Wood, is making a special appeal for new annual subscribers, and sincerely hopes that the public will respond generously. Contributions may be sent to him at the Hospital for Consumption, Fulham Road, S.W. 3.

**CHARING CROSS HOSPITAL.**—This hospital does nor fly the flag of distress. It has not issued an "S.O.S.," for it is not a "sinking ship" but one going "full steam ahead." Its work on behalf of suffering humanity is increasing every year. To keep pace with the demand, a new Nurses' Hostel has been secured, equipped and occupied. A new Maternity Department and new Children's Ward are in course of erection. These additions, which are vitally necessary, will mean an increase of £10,000 in the yearly expenditure. The policy of Charing Cross Hospital is to work quietly and unostentatiously and not to indulge in flag days, processions, bazaars and similar functions; to merit the generosity of its supporters by giving the greatest benefit to those who need its help, and to economise in every legitimate direction so as not to run into debt. If the reader approves of this policy, will he kindly show it in a practical manner by sending a donation, however small, to the House-Governor, Charing Cross Hospital, W.C. 2?

**CITY OF LONDON HOSPITAL FOR CHEST DISEASES.**—Nearly 1 in 10 die annually from consumption and 1 in 7 from heart disease. The work of this hospital in combating these diseases needs steady support, especially in view of the fact that the war increased the suffering from consumption. Help is required in order to meet an increase in expenditure of £15,000 per annum. The Secretary is Mr. George Watts, City of London Hospital for Chest Diseases, Victoria Park, E. 2.

**GUY'S HOSPITAL.**—Founded in 1724 by Thomas Guy, this great institution has to-day no less than 614 beds, and last year treated 9,653 in-patients, and 118,438 out-patients, whose attendances numbered about half-a-million. The annual cost of maintenance is £160,000, of which nearly £100,000 must be obtained by means of voluntary assistance. Subscriptions and donations are earnestly solicited by the Treasurer, Viscount Goschen, and should be addressed to him at Guy's Hospital, London Bridge, S.E. 1.

**KING'S COLLEGE HOSPITAL.**—During the year 1923, in spite of the restrictions due to the financial condition of the Hospital, it has been possible to make progress in two important directions. A powerful apparatus for Deep Therapy treatment has been installed at a cost of £1,500, which has been advanced upon loan. It is hoped to repay the amount by using the apparatus for private patients, but contributions have also been specially received for the apparatus. Towards the end of the year the new Dental School and a finely equipped Dental Department were opened under the auspices of the Medical School. The financial condition of the hospital has shown steady improvement, partly due to the use of the unoccupied wards for private patients. Arrangements made in this way have been much appreciated as placing hospital treatment at the disposal of a section of the community who are unable to afford a heavy expense and have prevented the closing of more wards to general patients With the cordial co-operation of the medical staff facilities have been provided for patients to make their financial arrangements with the House Governor of the Hospital, so that they may know the cost not only of their maintenance in hospital, but also of the medical fees before concluding arrangements for admission. The additional income, however, barely suffices to meet the expenditure, and further voluntary support is necessary before there can be any appreciable reduction in the debt of £70,000, which must be the preliminary to opening the wards to general patients. Donations should be sent to the Secretary, King's College Hospital, Denmark Hill, S.E.

**LONDON FEVER HOSPITAL.**—Established in 1802, the income is derived from voluntary sources, supplemented by patients' payments, which in the case of ward patients cover only a small proportion of the cost. During the year 1922, 814 patients were treated, all suffering from infectious diseases. Further help is needed to assist this, one of London's oldest charities, to carry on the work, of which the annual cost is about £25,000, and at least £10,000 of this sum must be collected in the New Year. Subscriptions and donations should be sent to Commander T. J. Farrell, D.S.C., Secretary, London Fever Hospital, Islington, N. 1.

**LONDON HOMŒOPATHIC HOSPITAL.**—Strong efforts have been made during the year by the London Homœopathic Hospital, Great Ormond Street, W.C. 1, with the support of its patron, the Duke of York, to increase its annual subscription list from the present inadequate amount of £2,000 to £3,000 in order to meet the demands of a daily growing number of patients, totalling in round numbers, during this year, to some 58,000 out-patient attendances and 1,700 in-patients. Towards the £1,000 required, the hospital has already raised £600, and a strong appeal is now made for a quick and generous response in the limited time that now remains to complete the list of new subscribers for presentation to H.R.H. the Duke of York at the close of the year. The Secretary is Mr. E. A. Attwood, London Homœopathic Hospital, Great Ormond Street, W.C. 1.

**LONDON HOSPITAL.**—The splendid work done by the London deserves and needs widespread support. The cost of its maintenance is tremendous—£256,992—and the public is aware that every penny subscribed is well and wisely used. Mr. E. W. Morris, the House Governor, will be grateful for subscriptions and donations, which should be sent to him at the Hospital, Whitechapel Road, E. 1.

**LONDON LOCK HOSPITAL.**—Nearly £10,000 is required by Christmas in order to carry on this work of national importance. Owing to the special ante-natal treatment available, 368 babies have been born free of venereal disease and thus given an opportunity of growing up strong and useful citizens. Donations should be sent to Mr. Henry J. Eason, F.C.I.S., London Lock Hospital, Harrow Road, W. 9.

**METROPOLITAN HOSPITAL.**—Situated in one of the poorest districts of London this hospital is urgently in need of financial help. In addition to this, funds are wanted for a new nurses' home, most urgently required for the proper accommodation of the staff, and special donations are solicited for the carrying out of this important and necessary work. Subscriptions and contributions should be addressed to the Treasurer or to Mr. Herbert F. Rutherford, secretary, Metropolitan Hospital, Kingsland Road, E. 8.

**ROYAL FREE HOSPITAL.**—To extend the existing buildings on an adjoining site recently presented to the hospital, £200,000 is required; the intention of the authorities being to provide extended accommodation for the treatment of patients as well as for undergraduate and post-graduate medical education. Grants and the allocation of funds for this object are specially sought. All contributions should be sent to the Secretary, Mr. Reginald R. Garratt, Royal Free Hospital, Gray's Inn Road, W.C. 1.

**ROYAL NORTHERN GROUP OF HOSPITALS.**—This group comprises the Royal Northern Hospital (Holloway), the Royal Chest Hospital (City Road), and Hospital of Recovery at Southgate and a Convalescent Home at Clacton. In spite of rigid economy, and the closing of seventy beds, the accumulated debt is now nearly £50,000, and the sick poor of North London are thereby deprived of much help and are suffering untold hardship. Assistance is needed at once, and needed badly, and the Secretary, Mr. Gilbert G. Panter, Royal Northern Hospital, Holloway, N. 7, will be most grateful for contributions.

**ST. GEORGE'S HOSPITAL.**—Over 30,000 patients are treated annually at this hospital, in addition to the 800 received at the convalescent branch at Wimbledon, and the committee most urgently appeal for Christmas donations to help towards the reduction of the deficit on the current year's work. In addition, contributions are earnestly desired for the extension of the Out-Patient Department, the work upon which has already been started, as increased accommodation is of vital necessity. The cost of this will be about £16,000, and any amount, large or small, will be thankfully received by Mr. James M. Churchfield, Secretary-Superintendent, St. George's Hospital, Hyde Park Corner, S.W. 1.

**ST. MARY'S HOSPITAL.**—The outstanding need of this well-known institution is the reduction of its formidable overdraft, which now stands at something like £30,000. Contributions to this end should be sent to the Secretary, Mr. W. Parkes, St. Mary's Hospital, Paddington, W. 2.

**SEAMEN'S HOSPITAL SOCIETY.**—This hospital, commonly known as the "Dreadnought," which now consists of five establishments, treated in the wards of the various Institutions 4,284 In-Patients during the year 1922, and over 16,000 Out-Patients, a total of 20,963. The Society comprises the Dreadnought Hospital at Greenwich, the Albert Dock Hospital, Connaught Road, the Hospital for Tropical Diseases, Endsleigh Gardens, King George's Sanatorium for Sailors, Bramshott, Hants., and the Angas Convalescent Home at Cudham, in Kent. Now the Society has been approached with a view to taking over the Tilbury Cottage Hospital. This hospital is situated in the dock district of Tilbury, and with the extension of these docks it is felt that there should be better facilities for the care and treatment of sick and injured seamen arriving in the great liners entering the port of Tilbury. The Society will take over this new responsibility with the start of the year 1924, and they feel confident that a sympathetic and generous public will continue to aid in affording succour to these men who face such dangers and endure such hardships to bring to these shores not only the luxuries but the necessaries of life. The Secretary is Sir James Michelli, C.M.G., Seamen's Hospital Society, Greenwich, S.E.

**UNIVERSITY COLLEGE HOSPITAL.**—The annual income of this hospital falls short of the expenditure by some £14,000, and, in the absence of any emergency grant or other unexpected receipt, the hospital authorities will have seriously to consider the question of curtailing their work. Last year over 5,300 in-patients and over 67,000 out-patients were treated. Subscriptions and donations are earnestly requested to enable the Committee to carry on the work of the Hospital. Mr. J. Gerald T. Buckle, Secretary, University College Hospital, W.C. 1.

**WESTMINSTER HOSPITAL.**—During the year 1922 2,473 in-patients and 14,504 out-patients were treated at this institution. To carry on the work on its present basis £40,000 is required annually, of which there is an assured income of only £20,000, leaving a balance of £20,000 to be obtained from voluntary contributions. The Hospital has been closed since July 1, 1923, for alterations and extensions which have become urgent and essential. Out-patients are being treated at the Medical School at Caxton Street, three minutes from the Hospital. Towards the £70,000 required for the reconstruction, £30,000 has been received or promised, and the Committee earnestly appeal for the remainder. Donations should be sent to the Secretary, Westminster Hospital, Broad Sanctuary, S.W. 1.

### HOSPITALS WITH 50 AND LESS THAN 150 BEDS.

**THE CANCER HOSPITAL (FREE).**—This is the only hospital in London solely devoted both to treatment and to research into the causes of cancer. Cancer is now generally recognised as one of the most prevalent and dreaded of human diseases. The Cancer Hospital (Free) therefore has outstanding claims upon the consideration of the benevolent public; and it is hoped that generous help may continue to be forthcoming to support this most worthy of institutions. Donations are also sought for the Reconstruction Building Fund, which is to provide new theatre accommodation and other improvements for the relief and benefit of the patients. Contributions should be sent to the Secretary, Mr. J. Courtney Buchanan, C.B.E., The Cancer Hospital (Free), Fulham Road, S.W. 3.

**CHELSEA HOSPITAL FOR WOMEN.**—The building of the greatly needed Nurses' Home is making good progress. A sum of £14,000 is still required. This work is supported by the King Edward's Fund, and when completed will free for patients some forty-five beds in the hospital now being used for the staff. It will be possible to use some of these beds for a class of patients for whom there is all too little provision, viz., those who through reduction of income can no longer pay the charges of nursing homes. Contributions towards the Building Fund or towards daily maintenance will be gratefully received by the Honorary Treasurer or by the Secretary, Mr. Herbert H. Jennings, at 44, Arthur Street, Chelsea, S.W. 3.

**CITY OF LONDON MATERNITY HOSPITAL.**—This is the oldest maternity hospital, and was established in 1750. When the present building was erected in 1908 ante-natal work at this hospital was in its infancy, but the institution is now devoting special interest to this branch, which has involved an expenditure of some £11,000. The present debt at the bankers, is £18,000, and a very strong appeal is made for generous donations towards the liquidation of this liability. Contributions should be addressed to the Secretary, City of London Maternity Hospital, 102 City Road, E.C.

**CLAPHAM MATERNITY HOSPITAL.**—This hospital though not in debt, earnestly appeals for Christmas donations or new subscribers to maintain and increase its usefulness. It is staffed by medical women, and its Hon. Secretary is Miss M. Ritchie, Clapham Maternity Hospital, S.W. 4.

**EVELINA HOSPITAL FOR CHILDREN.**—"The Evelina" is the largest hospital exclusively for children in the whole of south and south-east London, and serves several thickly populated and very destitute districts; it is for children of the poor only. Unfortunately, owing to its position, it is rarely seen by the "well-to-do" and suffers accordingly. A large sum is owing to the bankers, and contributions are needed to clear off the deficit; this year the expenditure has been unusually heavy, as the hospital has been entirely re-painted and renovated, and several important improvements and alterations have at the same time been carried out. The total expenditure has been about £3,000. All of this work was long overdue and had been delayed owing to the serious scarcity of funds, coupled with the War conditions. The ordinary expenditure is over £12,000, but the assured income falls far short of this sum, and about £8,000 has to be raised annually in voluntary contributions. The Secretary is Mr. H. C. Standiland Smith, Evelina Hospital for Children, Southwark, S.E. 1.

**EVERSFIELD CHEST HOSPITAL, ST. LEONARDS.**—A mortgage debt of £1,500 encumbers this hospital, well known for its excellent work. Many of the male patients are "war-broken" men who contracted consumption while defending the country. The hospital has therefore a special claim upon public sympathy, and, since the debt has been reduced from £4,500, it would be particularly grievous should friends fail speedily to clear off this remaining sum so that the institution may go forward unfettered.

**HAMPSTEAD GENERAL AND NORTH-WEST LONDON HOSPITAL.**—The expenditure of the Hospital has been reduced to between £21,000 and £22,000, probably the minimum that can be anticipated in view of prevailing conditions. Ordinary sources are producing an income at the rate of about £19,000 a year, which includes voluntary contributions by patients amounting to about £4,000. A debt of over £9,000 which so long hampered the Hospital was paid off from the proceeds of a very successful Festival Dinner held last June. Thus it remains only to raise each year an additional £3,000 to keep the Hospital free from debt. To this end the Council of the Hospital appeal to those who have not hitherto realised the need to contribute something each year towards the deficiency and thus enable them to carry on the work of the Hospital. The fact should not be lost sight of that the expenditure referred to makes no provision for those improvements and extensions which the advances of medical science and the increasing needs of the community demand. Contributions may be sent to the Secretary, Mr. Harold Wigg, Hampstead General and North-West London Hospital, Haverstock Hill, N.W. 3.

**LONDON TEMPERANCE HOSPITAL.**—The jubilee year of the London Temperance Hospital finds this institution in sore need of generous help. The premises occupied by the nursing staff have been, with other parts of the hospital, condemned by the Visiting Committee of the King Edward's Hospital Fund. A new Nurses' Home is therefore absolutely imperative. Many other improvements must be realised, notably in the operation department, to bring the hospital into line with modern requirements and to meet the needs ever pressing up to our doors. All donations should be sent to the Secretary, London Temperance Hospital, Hampstead Road, N.W. 1.

**MILLER GENERAL HOSPITAL FOR SOUTH-EAST LONDON.**—The extension of this hospital, which has been under consideration for some time, has now been definitely decided upon, and it is hoped that it will be possible to start building operations in the Spring of next year. The part of the scheme to be proceeded with is the first section of a much needed nurses' Home and one ward block, which will contain 3 wards of 26 beds each—one of which will be "the Borough of Deptford's War Memorial Children's Ward." The site has been paid for and about £20,000 is in hand, but a large further sum will be required. The Secretary is Mr. H. A. Bone, Miller General Hospital, Greenwich Road, S.E. 10.

**MOUNT VERNON HOSPITAL FOR TUBERCULOSIS.**—At the out-patients' department, Fitzroy Square, W., this hospital treats all deserving persons suffering from tuberculosis and diseases of the lungs and heart, and at the hospital at Northwood patients are admitted on a contributory basis. The Secretary, Mr. W. J. Morton, earnestly appeals for subscriptions and donations to carry on the good work which this institution is doing on behalf of the suffering poor. All help of a financial character should be addressed to him at the Mount Vernon Hospital, 7, Fitzroy Square, W. 1.

**PRINCE OF WALES'S GENERAL HOSPITAL.**—Tottenham is one of the poorest districts of Outer London, and this institution requires annually £23,000 to maintain its 125 beds. Over 30,000 patients are treated each year, and are received without subscribers' letters, the only passport to secure the services of the hospital being necessity. There is a debt at the bankers of £10,000, and an urgent appeal is made for donations towards the liquidation of this sum, which it is hoped will be forthcoming before the end of the year. Mr. F. W. Drewett, F.C.I.S., Director, Prince of Wales's General Hospital, Tottenham, N. 15.

**QUEEN CHARLOTTE'S LYING-IN HOSPITAL.**—Nearly 2,000 women are received into the wards of this institution every year, and over 2,000 are attended by the hospital midwives and nurses in their own homes. In addition, some 5,000 patients attend the ante-natal and child welfare department. In the midwifery training school about 150 students are trained in midwifery annually, and nearly 200 women are trained as midwives and maternity nurses. Great efforts have been made to free the hospital of debt, but nearly £20,000 is still required. Moreover, the much needed enlargement of the hospital, which was about to be commenced on the outbreak of war, has had to be postponed until more prosperous times. Donations will be gratefully received by the Secretary, Mr. Arthur Watts, at the Hospital, Marylebone Road, N.W. 1.

**THE QUEEN'S HOSPITAL FOR CHILDREN, HACKNEY ROAD.**—The outstanding need of this hospital is enlargement. Every department is worked up to the hilt. The institution is deservedly popular throughout the crowded districts that surround it, and it receives a great deal of support in the form of small contributions. Its expenditure has increased from £15,000 pre-war to about £31,000, and that of its branch, The Little Folks' Home, Bexhill, from £1,500 to £3,000. As its endowments only bring in £1,500 a year, it is naturally always in need of funds for maintenance. The struggle for existence has for the past nine years stood in the way of development, and a comprehensive scheme of enlargement, which early in 1914 had received the blessing of King Edward's Hospital Fund, has had to be put aside. The present accommodation for in-patients is limited to 170 beds (134 at Hackney Road and 36 at the branch establishment at Bexhill). With 40,000 children passing through its out-patient and casualty departments in the year (and an attendance of over 100,000 a year) these beds are, of course, insufficient. Lord William Cecil, the Chairman, and his Committee are asking for £20,000 this year to pay arrears and generally to improve their hospital's financial position.

**QUEEN MARY'S HOSPITAL FOR THE EAST END.**—This Hospital appeals to all interested in the work of looking after the sick and suffering. It serves a great part of the East-End of London with a teeming population, and is situated in one of the largest boroughs in England. Contributions should be sent to Major Raphael Jackson, Secretary, Queen Mary's Hospital for the East End, Stratford, E. 15.

**ST. MARK'S HOSPITAL FOR CANCER.**—The Committee of Management earnestly appeal for contributions towards £15,000 required for the enlargement of the hospital to cope with the increasing number of patients requiring alleviation from their sufferings. Additional wards, a new and more up-to-date out-patients' department, and larger cancer research laboratories are urgently required. Donations will be received by the Secretary, St. Mark's Hospital, City Road, E.C. 1.

**ST. PETER'S HOSPITAL.**—A sum of £2,500 is being asked for to improve the sleeping quarters of the domestic staff and to provide and equip an X-Ray Department. Contributions should be sent to Mr. Irwin H. Beattie, Secretary, St. Peter's Hospital, Henrietta Street, Covent Garden, W.C. 2.

**SAMARITAN FREE HOSPITAL FOR WOMEN.**—The need of a sum of £2,700 for essential repairs to the main fabric—both internal and external—of the hospital is most urgent, and the Governors are gravely concerned by the fact that through lack of funds they are unable to keep the hospital in a safe condition. A further cause of worry is the knowledge that by delaying these repairs the danger is intensified; every day the structural defects are becoming more serious, and the cost of repairing them will be much greater than the amount now estimated. Princess Arthur of Connaught writes: "How good this work is I know personally, not only as its President, but as having worked myself in the operating theatre and wards. I can testify to the splendid results obtained, and know that the money given to this hospital gives life and health to hundreds of poor women every year." Cheques should be made payable to The Treasurer, Samaritan Free Hospital, Marylebone Road, N.W. 1.

**SOUTH-EASTERN HOSPITAL FOR CHILDREN.**—To maintain efficiently its 60 cots, which are constantly occupied, this hospital began the year with a debt to bankers of £1,100, and an appeal is made for donations to free the institution of this liability. Contributions also are needed towards the rebuilding of the out-patients' department, which is inadequate to accommodate the 25,000 out-patients treated annually. Help towards these objects should be sent to Mr. W. Mason, Hon. Secretary, 88, Dacres Road, Forest Hill, S.E. 23.

**SOUTH LONDON HOSPITAL FOR WOMEN.**—The extension of the In-Patient Department by the adaptation of an adjoining building presented for this purpose so as to provide 36 additional beds for surgical cases will be opened to patients early in the New Year. The Building Fund of £12,000 is exhausted, and an amount of £3,000 has yet to be raised to meet charges under this head. A generous donor is providing a large proportion of the equipment, but household necessaries, including linen, will entail a further expenditure of £1,000. Donations towards this capital outlay will be most gratefully received. Especial appeal is made for new annual subscriptions and donations to meet the increased ordinary annual expenditure. Contributions should be sent to the Secretary, South London Hospital for Women, South Side, Clapham Common, S.W. 4.

**VICTORIA HOSPITAL, CHELSEA.**—For the year 1922 the institution again proved to be one of the most economically managed children's hospitals in London. As a result of special grants from the combined appeal and the Voluntary Hospitals Commission, the hospital debt had been reduced to £4,238 by December 31st. The ordinary income, however, is still insufficient to meet the yearly expenditure, and new annual subscriptions and donations are urgently required to place the institution on a better financial basis; 180 beds have to be maintained, including 50 at the Victoria Home, Broadstairs, the hospital's seaside branch. Contributions should be sent to the Secretary, Mr. D. St. John Bamford, Victoria Hospital for Children, Tite Street, Chelsea, S.W. 3. A new feature of the institution is the private nursing home for children recently opened at 29, Tite Street, adjoining the hospital. A great opportunity is now offered to parents who wish their children to have the comforts of a private home combined with the treatment and other advantages of a leading London Hospital.

---

### HOSPITALS WITH LESS THAN 50 BEDS.

**BELGRAVE HOSPITAL FOR CHILDREN.**—The new south wing is nearing completion, and will be ready for the admission of patients early in the New Year. In this wing there will be two additional wards containing twelve cots each, bringing the total number of available cots in the hospital up to 74. The Committee estimate that these extra beds will cost about £2,500 per annum to maintain, and they appeal for increased support in order that all the beds may be available for the sick children of South London without delay. Two thousand new annual subscribers are wanted. The Secretary is Mr. Thomas Clapham, Belgrave Hospital for Children, 1, Clapham Road, S.W. 9.

**MILDMAY MEMORIAL HOSPITAL.**—Owing to the drain on resources caused by the erection of fire staircases and balconies last year, this hospital has had to borrow funds to carry on the work which has greatly increased. Donations are thus urgently required and should be sent to the Secretary, Miss C. A. Lampitt, Mildmay Memorial Hospital, Newington Green Road, N. 1.

**PADDINGTON GREEN CHILDREN'S HOSPITAL.**—The hospital has 46 beds, and a Convalescent Home at Slough with 24 beds. The income for 1922 from all sources was £10,660, and the expenditure £11,876. Some 750 in-patients received treatment during the year, in addition to 11,498 new out-patients, while the total out-patients' attendances numbered about 40,000. The committee of management urgently appeal for new subscriptions, donations and legacies, to enable them to discharge the very heavy debt of £13,000 owing to bankers. Contributions will be thankfully received by the secretary, Mr. James A. Hamlin, at the Paddington Green Children's Hospital, W. 2.

**ROYAL EYE HOSPITAL.**—This is the only eye hospital in South London, and serves the needs of a very large and populous district. It has 22,000 new out-patients, more than 50,000 attendances annually, and its expenditure exceeds income by £1,000. Funds are urgently needed and should be sent to the Secretary, St. George's Circus, Southwark, S.E. 1.

**ROYAL WESTMINSTER OPHTHALMIC HOSPITAL.**—This hospital, founded 107 years ago, is greatly in need of funds to cope with the rapidly increasing work. As 84 per cent. of the required annual income has to be obtained from voluntary sources, constant appeal for support is necessary. During this year nearly 14,000 new patients, coming from all parts of the metropolis, have received treatment, representing upwards of 33,000 attendances. Contributions will be gratefully acknowledged by the Chairman or Secretary, at the Hospital, King William Street, West Strand, W.C. 2.

**ST. JOHN'S HOSPITAL FOR DISEASES OF THE SKIN.**—Founded in 1863, this hospital is the largest of its kind in the kingdom, dealing with 1,000 cases a week. Like other institutions, it is suffering from the present high prices of all commodities, and the board of management of this thoroughly deserving and necessary charity appeal earnestly for help. The recently formed London School of Dermatology is the recognised centre of dermatological teaching in London, its staff of Lecturers consisting of the Dermatologists from the twelve principal Medical Schools. Cheques towards the general expenses of the Hospital or for research work in Skin and Cancer will be gratefully received by the Treasurer, Captain E. C. Eric Smith, M.C., 49, Leicester Square, W.C. 2, or by the Secretary, at the same address.

### GENERAL CHARITIES.

**CHRISTMAS COMFORTS FOR PENSIONER PATIENTS.**—The Order of St. John and the British Red Cross Society have arranged, through the Ministry of Pensions, as in previous years, to provide additional comforts, such as cigarettes, tobacco, chocolate and games, for the men actually in Ministry Hospitals on Christmas Day. There are, however, ex-service pensioners who will be patients in other hospitals as a result of their war disabilities, and the Joint Council of the two bodies is endeavouring to get in touch with these hospitals, so that no eligible cases in hospital on Christmas Day may fail to receive the same gifts as those in Pensions Hospitals.

**DR. BARNARDO'S HOMES.**—The Charter of the Homes is "No Destitute Child Ever Refused Admission." The one and only qualification, therefore, for admission is destitution. In 1922, 13,449 boys and girls were dealt with; 1,351 being permanently admitted, and there are to-day 7,308 children under the care of the Homes. Some 28,382 boys and girls have been emigrated within the Empire, of which 98 per cent. are doing well. The institution comprises 156 separate branches and households, and the work formerly carried on at Her Majesty's Hospital, Stepney Causeway, is now transferred to the Australasian Hospital at the Girls' Village Home, Barkingside, Essex, where there are 1,400 girls always in residence; and to the John Capel Hanbury Hospital at the Boys' Garden City, Woodford Bridge, Essex, where are housed over 700 boys. Funds are urgently needed to support the work of this national charity, since, owing to the abnormal economic conditions prevailing since the war and the increase of unemployment, the demands upon Barnardo's resources are greater than ever. An earnest appeal is made for help, and will be welcomed at the Head Offices and Chief Ever-Open Door at 18-26, Stepney Causeway, E. 1.

**IMPERIAL CANCER RESEARCH FUND.**—This society is carrying on a very important work in the interest of suffering humanity, and it is hoped that its investigations into the causes of cancer, and its efforts to find a cure for this dreaded disease, may not be hampered for want of adequate funds. Donations and subscriptions should be sent to the honorary treasurer, Imperial Cancer Research Fund, 8-11, Queen Square, Bloomsbury, W.C. 1.

**HOSPITAL SUNDAY FUND.**—This Fund is appealing on behalf of the twenty-three hospitals and medical charities of Manchester and Salford. These form a suitable centre of healing, and the ordinary year's expenditure is as great as £340,000. The deficit of the year is £52,433, and all contributions should be sent to the Secretary, Capt. T. Spencer, 94, Market Street, Manchester.

**MENTAL AFTER-CARE ASSOCIATION.**—Seven thousand men and women are discharged every year from our mental hospitals, and a number of these have no home, no friends and no financial resources. It is to help these unfortunate people that this Association exists, since, unaided, many relapse and become permanent burdens on the rates or sink into abject poverty. The Association, whose Secretary is Miss Vickers, Church House, Westminster, S.W., acts as a Labour Bureau, finding not only occupation, but often the means necessary to undertake it, by clothing, tools, and in other ways.

